# Toward an integrative neurovascular framework for studying brain networks

**DOI:** 10.1117/1.NPh.9.3.032211

**Published:** 2022-04-07

**Authors:** Jérémie Guilbert, Antoine Légaré, Paul De Koninck, Patrick Desrosiers, Michèle Desjardins

**Affiliations:** a Université Laval, Department of Physics, Physical Engineering, and Optics, Québec, Canada; b Université Laval, Centre de recherche du CHU de Québec, Québec, Canada; c Centre de recherche CERVO, Québec, Canada; d Université Laval, Department of Biochemistry, Microbiology, and Bioinformatics, Québec, Canada

**Keywords:** neurovascular networks, functional connectivity, neurovascular coupling, vasculo-neuronal interactions, BOLD fMRI, brain optical imaging

## Abstract

Brain functional connectivity based on the measure of blood oxygen level-dependent (BOLD) functional magnetic resonance imaging (fMRI) signals has become one of the most widely used measurements in human neuroimaging. However, the nature of the functional networks revealed by BOLD fMRI can be ambiguous, as highlighted by a recent series of experiments that have suggested that typical resting-state networks can be replicated from purely vascular or physiologically driven BOLD signals. After going through a brief review of the key concepts of brain network analysis, we explore how the vascular and neuronal systems interact to give rise to the brain functional networks measured with BOLD fMRI. This leads us to emphasize a view of the vascular network not only as a confounding element in fMRI but also as a functionally relevant system that is entangled with the neuronal network. To study the vascular and neuronal underpinnings of BOLD functional connectivity, we consider a combination of methodological avenues based on multiscale and multimodal optical imaging in mice, used in combination with computational models that allow the integration of vascular information to explain functional connectivity.

## 1 Introduction

Although the brain has long been recognized as an intricate network of neurons rather than a collection of segregated processing units, only the arrival of noninvasive brain imaging techniques in the last decades has allowed neuroscientists to gaze at the large-scale network structure of the human brain. The most popular of these techniques, blood oxygen level-dependent (BOLD) functional magnetic resonance imaging (fMRI), notably allows us to infer neuronal activity in the entire brain by measuring blood oxygenation changes that occur through neurovascular coupling.^1^^–^^3^ While BOLD fMRI has been critical to the application of network neuroscience to study the human brain, a fundamental limit of the approach is its inability to observe neuronal networks directly, as it must rely on the brain vasculature as an intermediary to probe neuronal systems. Even though the coupling between neurons and blood vessels is a local effect that is usually described at the cellular scale, we highlight here how vasculature and hemodynamics can alter our fMRI-based representations of large-scale distributed functional brain networks in humans. We start with a brief overview of the concept of functional connectivity (FC) that is now widespread in the current literature on human brain networks. We then go on to revisit how hemodynamic effects influence measurements of FC and doing so review evidence for the emerging hypothesis of overlapped vascular and neuronal functional networks.^4^^–^^6^ While numerous methods have been developed to correct vascular effects in fMRI, essentially treating them as confounds to neuronal signals,^7^^–^^9^ we also focus here on viewing the vasculature as a synergistic element actively involved in shaping neuronal dynamics over diverse time scales, causing BOLD fMRI to effectively capture entangled neurovascular networks.^9^ We end by an exploration of various experimental tools and models of connectivity that could eventually help us to better understand the neuronal and vascular underpinnings of FC. Our goal with this review is to highlight some of the caveats associated with BOLD-derived FC measurements and to stimulate ideas aimed at elucidating how complex neurovascular interactions shape human brain functional networks.

## 2 Network Approach to Studying the Brain

The study of brain connectivity and networks has become a standard paradigm in neuroscience research, in line with the modern view of the brain as a single, complex system.^10^ The brain can be modeled as a complex network of interconnected neurons using approaches based on graph theory.^11^ Graphs are mathematical structures which represent discrete sets of pairwise interactions between objects, represented by edges and nodes, respectively.^12^ A quick glance at a graph representation is evocative of neurons and their synaptic connections (Fig. 2), leading to an intuitive application of graph theory to neuronal systems. Noninvasive neuroimaging techniques such as fMRI cannot, however, distinguish the fundamental neuronal units of brain networks, being spatially limited to millimeter-sized voxels in which millions of neurons are densely packed. Connectivity and network interactions can nonetheless be observed across multiple scales, as whole-brain neuroimaging data capture macroscopic and mesoscopic networks of interconnected neuronal populations with complex functional interactions.^13^ As such, at the larger scales that are characteristic of human neuroimaging data, nodes are typically defined as groups of gray matter voxels, based on anatomical boundaries or parcellations obtained from brain-mapping techniques.^14^ The edges between nodes for their part can represent either structural or functional interactions.

### 2.1 Structural and Functional Connectivity

At a fundamental level, structural connectivity (SC) designates connections that are formed by a physical substrate between nodes. In brain networks, this occurs when two nodes are connected synaptically, but the nature of the connection depends on the scale that is considered. For instance, at cellular resolution, structural links could represent individual synapses, while at the mesoscale they may represent myelinated axonal projections between brain regions.^15^ Anatomical projections can be measured noninvasively in humans using diffusion-weighted imaging (DWI), in which signal is generated from water molecules diffusing along white matter tracts. Structural edges are then inferred from DWI scans using tractography algorithms.^16^^,^^17^ A fundamental limitation of DWI is its inability to resolve edge direction in SC, thus leading to undirected graphs, which do not directly inform about the causal influence that nodes exert on each other. Directionality in SC may be inferred in animal models from postmortem imaging techniques, for instance from reconstruction of electron microscopy slices, where pre- and postsynaptic elements are distinguishable,^18^ or by tract tracing, in which a fluorescent tracer is injected in a specific part of the brain and diffuses either anterogradely or retrogradely along axonal projections.^19^^,^^20^

The interaction between structurally connected nodes leads to the emergence of network dynamics, which can be described using an alternative form of connectivity. FC is defined as a measure of the statistical codependency between activity measurements in different node locations. In brain networks, this definition rests upon the assumption that cofluctuating areas are likely to be either communicating directly^21^ or driven by shared inputs and thus to be involved in similar functions. Activity can refer to direct measures of neuronal activity or proxies from electrical (e.g., EEG), optical (e.g., fNIRS), or magnetic resonance recordings. The nature of the functional relation between nodes can again be directed (causal), as inferred from the temporal lags between nodal time series^22^^,^^23^ or modeling frameworks such as dynamic causal modeling (DCM).^24^^,^^25^ Alternatively, it can be undirected, as determined by correlation measures. Although we will return to causal connectivity later, we will next focus on undirected FC typically characterized by the Pearson correlation coefficient between the time series of nodal activity.

### 2.2 Functional Networks

The term “functional brain network,” within the framework of network neuroscience, refers to a description of all interactions between regions distributed across the whole brain. However, subsets of brain regions, which have been reliably observed to be coactive across human subjects^26^ and species,^27^^,^^28^ are also commonly referred to as functional brain networks. Such groups of cofluctuating regions can be observed in both resting-state and task-evoked fMRI, in which case they are respectively called resting-state networks (RSNs) and task-positive (or negative) networks. They are associated with different functional roles, as exemplified by their activation in response to diverse cognitive states or demands. For instance, the default mode network, comprising regions of the prefrontal and cingulate cortex, precuneus, and inferior parietal lobules, is normally active when subjects are at rest, but goes silent when cognitive load increases.^29^ Multiple methods have been used to identify resting-state or task-responsive networks from neuroimaging data, the most widespread being seed-based correlation and independent component analysis (ICA).^30^ These different approaches have led to convergent spatial maps of coactive regions,^31^ such that many of them, like the default mode network, are now considered archetypal features of the brain. The regions that form an RSN or task-responsive network are highly interconnected both functionally and structurally^32^ and can be viewed as forming a network of their own, but from the whole-brain network perspective, they are modules of a larger network. As will be described later, new evidence suggests that these spatial footprints of neuronal activity could also be relevant to explain the brain’s complex vascular organization.

### 2.3 Neurovascular Coupling in Human Functional Networks

Due to the preponderance of fMRI in the human neuroimaging literature, the term “functional connectivity” has become chiefly defined by the study of correlations between regional or voxel-wise activity as measured with BOLD fMRI. In this article, we will use the term BOLD FC to refer to this definition, as opposed to the broader sense of FC defined above. BOLD FC is mostly used as a neuroimaging tool under the premise that hemodynamic measurements are underlaid by synchronized neuronal activity. This premise has been supported by many experiments, notably studies using combined fMRI and intracranial recordings in humans^33^^,^^34^ that have shown good agreement between spatial patterns of brain electrical activity and BOLD RSNs. Results from animal experiments have also shown that slow neuronal oscillations drive fluctuations in arteriole diameter in the same frequency band within which FC is evaluated in resting-state fMRI.^35^ However, other animal studies have also observed weak temporal correlations between spontaneous hemodynamic signals and electrophysiological recordings, for example, in the awake mouse barrel cortex during epochs of rest^36^ and in the anesthetized rat striatum, where the correlations are reduced even further when dopaminergic activity is enhanced.^37^ Such observations reflect the state and brain region dependency of neurovascular coupling and highlight the challenge of interpreting BOLD FC in strictly neuronal terms.^38^ The ambiguous nature of BOLD FC is further compounded by experiments that have observed the spatial signatures of typical RSNs from non-neuronal signals.^4^^,^^6^^,^^39^^,^^40^

Despite its possibly ambiguous interpretation, BOLD FC and its derived graph-theoretical metrics have shown great promise as eventual clinical markers.^41^^,^^42^ For instance, reliable group differences in FC-derived metrics have been measured in pathologies, such as autism,^43^ schizophrenia,^44^ Alzheimer’s disease,^45^^,^^46^ and in traumatic brain injury.^47^ However, from a treatment development perspective, it would be useful to identify whether such disease-related changes in BOLD FC are caused by neuronal, vascular, or mixed effects. This is particularly important given that numerous neurodegenerative and neurodevelopmental disorders are strongly associated and sometimes preceded by vascular irregularities (see Ref. 48 for a recent review). In the next section, we will review how various vascular parameters influence measures of FC and present evidence arguing that observations of archetypal functional networks from non-neuronal signals do not challenge the neuronal origin of these networks, but suggest that BOLD FC captures an overlapped representation of vascular and neuronal networks.

## 3 Vascular Influences on BOLD Functional Connectivity

Since whole brain functional networks are for the moment visible in humans only through the brain’s vasculature, it is crucial to have a quantitative understanding of how vascular structure and function influence BOLD FC measurements. We thus begin this section by reviewing how measurable neurovascular features such as the balance between cerebral blood flow (CBF) and cerebral metabolic rate of oxygen consumption (CMRO2), but also purely vascular properties such as blood vessel reactivity manifest themselves in BOLD FC measurements. We also take a slight detour to emphasize the relevance of combining vascular measurements with BOLD FC to characterize brain pathologies.

### 3.1 Interplay Between CBF/CMRO2 Coupling, SNR, and FC

BOLD FC is classically determined by computing the correlation coefficient between regional BOLD time series, although possible alternatives will be briefly discussed later. Under the view that neuronal connectivity is the variable of interest, any non-neuronal factor that influences BOLD correlations will thus confound the measured connectivity. One crucial factor in determining the outcome of correlation measurements is SNR, as two strongly correlated time courses can appear less correlated if random noise comes to dominate the “true” signals.^49^ With BOLD, signal amplitude is inversely related to the amount of deoxyhemoglobin (HbR) in a brain voxel. This is in turn related to two main time-varying quantities: HbR in, determined by CMRO2, and HbR out, determined by CBF.^50^^–^^53^ A positive BOLD signal occurs during neuronal activation when functional hyperemia increases CBF above energetic demands.^54^ It is thus the mismatch between CBF and CMRO2, or CBF/CMRO2 coupling, that largely determines BOLD SNR (i.e., the BOLD contrast amplitude over signal variance).^52^^,^^53^ Hence, a decrease in CBF/CMRO2 coupling can result in lower BOLD FC even in the presence of strong neuronal FC.^55^ This is important since potentially confounding changes in the relationship between flow and metabolism can occur in patients with various brain conditions^56^^–^^58^ and even in healthy individuals after caffeine consumption,^59^^,^^60^ during task-performance,^61^^,^^62^ or between different brain regions in the same subject.^63^^,^^64^

Apart from relative signal amplitude, the relative timing between CBF and CMRO2 is also key to the correlation of BOLD time courses between two regions. An increase in the arrival time of oxygenated blood in a vascular domain will dephase this domain’s initially synchronized BOLD fluctuations with other regions,^49^ lowering FC in a manner that depends on the underlying delay in oxygen consumption.^55^

### 3.2 CBF/FC Coupling as a Possible Neuroimaging Disease Marker

Given the role of CBF in fueling neuronal communication, its association with brain-wide FC metrics should come as no surprise. The presence of such an association can be verified by combining arterial spin labeling imaging with BOLD fMRI to measure perfusion and BOLD connectivity in the same imaging session. In such studies, a commonly reported connectivity metric is functional connectivity strength (FCS). FCS is a graph-theoretical measure of centrality and can be defined as the average connection weight (correlation coefficient) between a node and all other network nodes. In brain networks, nodes with high FCS are often associated with hubs, regions that integrate information from multiple segregated areas and thus form on average more numerous and stronger connections. In a study conducted on healthy subjects, Liang et al. identified a strong correlation between a node’s perfusion level and FCS.^65^ This relationship was distance dependent, as CBF was a better predictor of a node’s connectivity to remote than to nearby nodes, suggesting that the level to which a brain region is supplied with blood depends on its topological role within the network. Given that their higher perfusion level seems to reflect elevated baseline metabolism,^66^ it has been proposed that hub regions could be especially vulnerable to metabolic or activity-induced stress.^67^^–^^69^ Conversely, a failure by the vascular system to deliver sufficient energy to regions that theoretically play such a key role in information exchange could also be a hallmark of many brain disorders.

Studies published in recent years have accordingly found signs of altered CBF/FCS coupling in individuals with schizophrenia,^70^ Wilson’s disease,^71^ primary open-angle glaucoma,^72^ and white matter lesions.^73^ In Alzheimer’s disease patients, decreases in FC estimated with ICA were found to be linked to reduced perfusion, a relationship that was absent in healthy controls.^74^ Also in Alzheimer’s patients, Zheng et al. found specific regional disruptions in CBF and FC and proposed a biomarker based on CBF and BOLD low-frequency oscillations amplitude in the posterior cingulate cortex and left precuneus.^75^ Apart from CBF, other metrics, such as vascular volume fraction,^76^ have been used to investigate the relationship between BOLD FC and the brain vasculature and could eventually be used to add an observational dimension in the search of disease markers. Overall, these studies suggest that fMRI-derived graph-theoretical metrics, when combined with vascular measurements, can yield greater insights into pathological processes than when taken on their own, in line with the growing number of observed associations between the brain vasculature and the onset and progression of neurodegenerative diseases.^48^

### 3.3 Cerebrovascular Reactivity and FC

After seeing how the regional amplitudes and delays of CBF signals can affect measurements of BOLD FC, both being determinant to the observed correlation between two regions, we now ask what factor could potentially play a key role in establishing those region and subject-specific properties.

Regional CBF levels are strongly related to metabolically costly neuronal activity through neurovascular coupling.^29^^,^^65^ However, regional variations in vascular response amplitude and latency are also known to occur through neuronally independent means. To study vascular regulation and synchronization independently of neuronal (metabolic) contributions, we can turn to insights provided by studies of cerebrovascular reactivity (CVR). CVR measures the ability of blood vessels to actively dilate and constrict, and hence to control CBF, in response to a vasoactive stimulus. It is a purely vascular property and considered to reflect vascular endothelium and smooth muscle function. Its measure is often done by observing BOLD changes to varying arterial partial pressure of ${\mathrm{CO}}^{2}$, a vasodilator that globally increases arterial diameter via a NO-dependent pathway.^77^^,^^78^ Importantly, this method assumes that ${\mathrm{CO}}^{2}$ causes negligible metabolic/neuronal effects, which has been challenged.^79^^,^^80^ CVR mapping is notably used to separate metabolic from CBF contributions to the BOLD signal in calibrated BOLD experiments, and its importance in the interpretation of RSNs is increasingly being recognized (see the work by Chen and Gauthier^81^ for review).

A notable characteristic of vascular responses to vasoactive stimuli is their high spatial heterogeneity, both in terms of amplitude and dynamics. During breath-hold tasks used to globally increase ${\mathrm{CO}}^{2}$ arterial pressure, a difference up to 6 s in the time to maximal BOLD amplitude can be observed across the brain.^82^ The fact that different brain regions can respond to the same stimulus with varying delays has important implications for correlation-based FC. As CVR also dictates the strength of CBF responses to vasoactive stimuli, and thus indirectly of BOLD SNR, we could expect it to be a strong determinant of the ability to use BOLD to detect correlations between regions with functional neuronal connections. Accordingly, observational studies have shown that individuals with stronger CVR are more likely to display higher BOLD FC.^83^^,^^84^ This relationship can also be experimentally observed within individuals by manipulating baseline arterial CO_2_ levels, as hypercapnia leads to decreased reactivity.^84^^–^^86^ The result that ${\mathrm{CO}}^{2}$ inhalation results in lower BOLD connectivity^80^^,^^84^^,^^87^^,^^88^ is thus also consistent with the proposed influence of CVR on BOLD FC.

### 3.4 Physiological Correlations and FC

Another important contributor to BOLD FC is the regional level of physiological BOLD correlations. Such correlations are often depicted as noise that hides true neuronal correlations. An example is the propagation through the brain vasculature of low frequency oscillations that originate from outside the brain, described in studies by Tong et al.^4^^,^^89^ and reviewed in Ref. 90. Such oscillations can, for example, arrive in the brain through the carotid arteries and then split into both brain hemispheres, giving rise to symmetrical correlations that are independent of neural activity. The amount of such physiological “noise” in a region could potentially modulate the positive effect of CVR on BOLD FC, even negating it in regions where physiological contributions dominate neuronal ones.^80^^,^^83^

A key variable possibly affecting the importance of physiological noise is the regional level of vascularization. Blood vessel density is known to account for a substantial portion of the variance in resting-state BOLD amplitude,^91^ and FCS has been shown to be inversely proportional to the macrovascular volume fraction within a voxel.^76^ The strong vascularization of the occipital cortex^92^ has accordingly been suggested to explain the reduced sensitivity of BOLD FC in this region.^93^ It is thus plausible that in strongly vascularized regions, physiological noise domination could reduce the ability of BOLD FC to detect weaker neuronal correlations.

The evidence we reviewed suggests that structural vascular features such as vascular density, but also functional features such as the regionally varying responsivity of blood vessels captured by CVR, have a significant impact on the functional networks we can measure with BOLD fMRI. Traditional denoising strategies based on the use of global nuisance regressors are inappropriate in the presence of such spatial heterogeneity. Other techniques such as ICA, which decomposes imaging data in mathematically independent spatiotemporal patterns, have, however, shown a good ability to isolate experimental effects, neuronal and physiological contributions as well as artifacts such as those resulting from motion in fMRI.^94^^–^^96^

Furthermore, while the simplicity of the Pearson correlation coefficient makes it a statistical measurement of choice for inferring FC, it remains especially sensitive to the potentially confounding vascular influences on BOLD SNR and delays. Connectivity could alternatively be inferred from statistical quantities, such as cross-correlation, mutual information, Granger causality or transfer entropy, to name a few.^97^ Such higher-order statistical measurements could potentially be less sensitive to vascular artifacts. For example, while classical correlation analysis would miss the correlation between two synchronized neuronal signals that are translated to BOLD with different time delays, cross correlation would capture this information by considering correlations at multiple time lags. However, as we will emphasize, the occurrence of distributed vascularly or physiologically driven BOLD (de)synchronization could also reflect functionally relevant entangled neurovascular territories. In the following section, we review and discuss the implications of a series of recent experiments that have suggested that typically measured BOLD functional networks represent the overlap of functional networks of neuronal origins with functional networks generated through purely vascularly driven BOLD synchronization.

### 3.5 Overlapped Neurovascular Networks Hypothesis

In a recent study, Bright et al. showed in an elegant way how BOLD task-responsive brain networks might be synchronized through both neuronal and vascular mechanisms.^5^ To do so, they identified functional brain networks that were either activated or deactivated by a visual or working memory task, while intermittently presenting subjects with a vasodilatory stimulus (${\mathrm{CO}}^{2}$ inhalation). They then demonstrated that each of the identified task-dependent network had an associated spatially overlapped network, but whose activity was time-locked to the presentation of the vascular challenge instead of the sensory or cognitive stimuli. Thus, even though the effect of ${\mathrm{CO}}^{2}$ inhalation is supposed to be global, the responses to increases in arterial ${\mathrm{CO}}^{2}$ were spatially segregated in groups of regions that also happened to form a neuronal task-responsive functional network.

In another recent study, Chen et al. estimated the voxelwise BOLD responses to respiratory variation and heart rate changes.^6^ Using a clustering algorithm to parcellate the brain into regions with similar responses to these physiological signals, they showed that the resulting parcellation tended to organize the brain in modules that resemble classic RSNs.

A final revealing example involves the previously mentioned systemic low-frequency oscillations that propagate through the brain vasculature identified by Tong et al.^4^ In their study, the authors measured traveling oxygenation variations in the periphery (fingertips) and estimated the time required for this signal to reach each brain voxel. This information was then used to build a synthetic resting-state dataset representing the propagation of the signal through the brain vasculature. Despite the synthetic time courses containing only information about the time-shifts of a systemic vascular signal, the authors could extract from these data strong replications of archetypal RSNs. In follow-up studies, the temporal delays associated with this signal were shown to be consistent with a blood-born signal traveling along the vasculature.^89^^,^^98^

**Fig. 1:**
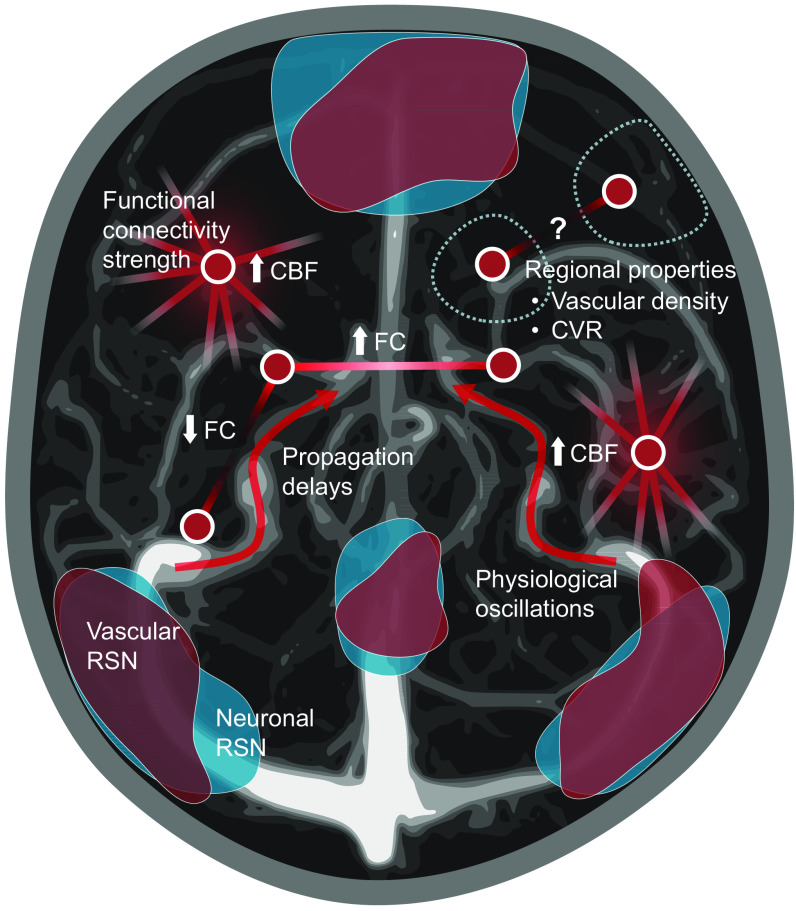
Vascular influences on BOLD FC. Functional connections observed through BOLD fMRI can be influenced in various ways by the vasculature. Nodes and edges depict elements of a BOLD functional network. Propagation delays of blood-borne signals can induce time lags which reduce correlations or generate spurious ones. Physiological oscillations can create non-neuronal correlations which may be accentuated in strongly vascularized regions such as the occipital cortex, making it harder to detect neuronal correlations. CBF is increased in hub regions where metabolic requirements are heightened, highlighting a local form of coupling between vascular and neuronal organization. Furthermore, vascular properties are thought to be organized in spatially remote areas to functionally match RSNs. The resulting coordinated delivery of blood in RSNs leads to observations in fMRI of spatial components associated with purely vascular signals in addition to neuronally-driven ones.

Overall, these studies show that the synchronization of BOLD fluctuations across the brain may be due to both (1) coherent neuronal activity and (2) coordinated oxygenation fluctuations that can be explained by physiological or vascular considerations alone (Fig. 1). The evidence we reviewed suggests that these include (without being limited to) the spatial distribution of vascular properties (e.g., CVR and vascular density), as well as the three-dimensional (3D) configuration of the vascular network. The latter could constrain blood-traveling signals that generate large-scale non-neuronal correlations causing apparent connectivity in fMRI studies. These would result in the observed vascular-regulated functional networks that mimic the spatial distribution of known neuronal functional networks. In addition to the reviewed experiments, this interpretation is also consistent with other observations of duplicated networks.^39^^,^^99^

The presence of coexisting neurovascular networks lends itself to an interesting evolutionary interpretation: while the network of neurons in the brain has evolved under the pressure of balancing the benefits of functional segregation (low wiring cost) with those of large-scale integration (high communication efficiency),^68^^,^^100^^–^^102^ it has also evolved under the need to ensure its various modules could constantly be supplied with sufficient energy according to their relative needs. Because of the system’s complex network organization and of finite energy resources, the vasculature co-evolved as an energy-delivering network that has adopted some functional and structural features that are spatially organized similarly to functional neuronal networks. By providing blood vessels in distant brain regions which operate synchronously with similar dynamic properties (which could be instantiated via the properties of pericytes, astrocytes or other CBF-regulating cells), the vascular system could form “vascular functional networks.” These would serve to coordinate blood delivery in the most efficient manner for the metabolic support of the entire complex neuronal network. In addition to the vasculature’s functional properties, its spatial configuration could also be formed to accommodate functional networks by properly uniformizing blood propagation delays in frequently coactive regions, explaining how the delay maps used by Tong et al. to build synthetic fMRI datasets could possibly contain neuronal information.^4^^,^^90^ During development, this could be orchestrated by the known bidirectional communication mechanisms between neurons and nascent blood vessels^103^ to ensure the formation of a dense capillary bed that spatially matches brain metabolic demands.^104^^,^^105^

The works we reviewed support a view of the vasculature as not only structurally or functionally tuned to local energy demands but also designed to support large-scale network organization of brain activity requiring spatiotemporally coordinated energy supply. This organization is reflected in vascular functional networks, a term we use to designate groups of regions whose coherent BOLD oscillations are driven by vascular sources independently of neuronal activity. With the application of common functional network identification methods (e.g., seed-based correlations, ICA), these give rise to replications of known neuronal functional networks.

### 3.6 Vasculoneuronal Interactions

Although vascular functional networks have for now been proposed to spatially match neuronal networks for metabolic support, another possible interpretation for their observation, as suggested by Bright et al.,^5^ is that they still fundamentally represent neuronal fluctuations, but that would be actively driven by the vasculature. This explanation relates to the so-called hemoneural hypothesis initially proposed by Moore and Cao.^106^ These authors, noting various ways in which hemodynamics could have an influence on neuronal activity, in reverse of the canonical neurovascular coupling direction, posited that blood flow may play an important role in information processing in the brain. Proposed hemoneural transduction mechanisms involve endothelial nitric oxide, which can affect neuronal polarization^107^ and synaptic plasticity,^108^ the cooling action of CBF and the associated influence of temperature on neuronal activity,^109^ the activation of mechanosensitive ion channels following vessel dilation and the modulation of neuronal processing by vascular-sensitive perivascular astrocytes. More recently, Kim et al. obtained evidence for these last two mechanisms by showing that increases in arterial tone could be sensed by perivascular astrocytes through the mechano-sensitive TRPV4 channel and that the subsequent increase in astrocytic calcium mediated a decrease in pyramidal neurons firing rate.^110^ The same TRPV4 channel has also been suggested to be recruited in an astrocytic feedback control mechanism of slow arteriole oscillations.^111^ Another line of evidence for vascular influence on neural activity is from observations of modified electromagnetic cortical activity during hypercapnia^79^^,^^80^ (these observations are the same that challenge the use of ${\mathrm{CO}}^{2}$ inhalation as a purely vascular stimuli). Recently, a study also proposed an interesting vascular feedback mechanism in the hypothalamus by which the firing of vasopressin neurons, acting to re-establish body fluid homeostasis after a sudden increase in systemic sodium levels, can be maintained over a prolonged period.^112^ After sensing increases in circulating sodium levels, the firing of this population triggers a slow release of vasopressin, which acts as a vasoconstrictor on nearby vessels. The ensuing decrease in blood flow produces a hypoxic environment that would serve as a positive feedback mechanism to maintain elevated neuronal firing rates and vasopressin release. Since much of the experimental work showing vascular to neuronal communication involves the use of brain slice models, it would be interesting to see more of these mechanisms investigated *in vivo*.

The possibility of hemoneural communication does not diminish the importance of already known neurovascular communication pathways, but the emerging literature on vascular feedback mechanisms suggests that different contexts may be better explained by a different combination of the two. For example, to describe rapid (∼s) flow increases in cortical responses to sensory stimuli, feedforward neurovascular coupling might be sufficient, while vascular modulation of neuronal activity in the resting-state to accommodate changes in systemic pressure might be best described by vasculoneuronal coupling.^110^ Context may also refer to the brain region and time scale at play, for example, when considering prolonged (∼h) hypothalamic responses aimed at maintaining homeostasis during a systemic challenge.^112^ If the early appearance of CBF reductions truly has a causal role to play in neurodegenerative diseases such as Alzheimer’s or schizophrenia,^113^^–^^117^ then the ensuing disruption of neuronal networks spanning multiple years is also likely to involve, at least initially, vasculoneuronal effects. Other indirect evidence of causal vascular influence on neuronal systems includes the impact of slow breathing and heart rate variability on FC in emotion regulation networks,^118^ the link between cardiovascular health, cognition, and structural brain changes^119^ as well as between age-related cognitive decline and cardiovascular risk factors.^120^^,^^121^

Does vasculoneuronal communication help describe observed neuronal and BOLD signal dynamics under certain conditions and time scales? Do these effects contribute to information processing? What are the respective contributions of neuronal and vascular compartments to both local BOLD signals and the coherence between spatially separated signals? To begin answering such questions about the physiological mechanisms underlying BOLD functional networks and brain function in general, we turn in the next section to recent experimental advances that allow measurements of neuronal and vascular-based networks in animal models at different scales (see Ref. 122 for an excellent review on the subject). Importantly, since macroscopic brain networks are made of microscopic cellular networks, we emphasize certain combinations of animal models and imaging techniques that together give access to a multiscale portrait of neurovascular interactions. We end by reviewing modeling approaches that can leverage such multiscale neurovascular imaging data to either provide more physiologically grounded and causal interpretations of BOLD FC or explain more variance in patterns of functional connections.

## 4 Toward the Disentangling of Neurovascular Networks

Let us consider the hypothesis that vascular structure and function can influence BOLD FC in at least two ways: (1) via the regional- and subject-specific hemodynamic filter through which neuronal signals are converted to BOLD signals, and (2) via their synergistic interactions with neurons, mediated by other cells of the neurovascular unit (NVU) which could directly shape neuronal dynamics. These influences roughly encapsulate the two (nonexclusive) ways in which vascular imprints on BOLD functional networks can be viewed: either as confounds or as functionally relevant features. In this section, we start by discussing imaging approaches that can help us see through or study those two types of influences. Toward the former, we review how recent optical methods are constantly bringing us closer to directly accessing neuronal dynamics and connectivity, without the filtering action of hemodynamics. We also highlight the current limits and technical challenges of extending them to the mouse, a translational model of choice. Toward the latter, we discuss how mouse studies can provide network measurements of both neuronal and hemodynamic signals at multiple spatial resolutions, providing a means to study their mutual influence and the mechanisms that unite them across observational scales.

### 4.1 Towards All-Neuronal FC Using Optical Techniques

In animals, neuronal activity can be directly measured without the proxy of fMRI by virtue of combining genetically encoded calcium indicators (GECIs) or contrast agents with fluorescence imaging techniques. Fluctuations in calcium indicator fluorescence report local calcium (${\mathrm{Ca}}^{2+}$) concentration, related to spiking activity, in the cells or subcellular regions where the indicators are targeted. The most widely used GECI, GCaMP, is still being continuously improved.^123^^,^^124^ Although the slow decay rate of calcium buffers makes calcium signals unable to clearly resolve individual spikes that are separated by less than ∼1  s, many inference methods have been proposed to recover the underlying spiking activity, given a fast enough sampling frequency.^125^^–^^129^ Voltage-sensitive dyes^130^^,^^131^ offer another avenue for measuring neuronal activity with high-temporal resolution using fluorescence microscopy and have been deemed “all-optical electrophysiology.” In naturally transparent organisms expressing GCaMP, such as *C. elegans* and the zebrafish larva, calcium activity can be recorded optically in neurons across the entire brain with a temporal resolution of ∼1 to 3 Hz using lightsheet microscopy^132^ or fast laser-scanning multiphoton microscopy (LSMPM).^133^ From these whole-brain neuronal recordings in small animal models, both functional and structural network approaches have led to descriptions of topological features that are similar to well-described features of BOLD FC in humans, such as a modular structure of interconnected regions in zebrafish^134^^,^^135^ and a regionally heterogeneous structure–function relationship in drosophila.^136^^,^^137^ While it is interesting to observe similarities across species and imaging modalities, supporting the neuronal origins of BOLD FC topological measurements made at much larger scales, all-neuronal FC in small fish and insects sheds little light on the complex neurovascular interactions in mammal brains.

In a translational perspective, a cross-species approach in which measurements from multiple species with the various imaging modalities that are available for each of them will be necessary if we hope to extract a maximum of neurophysiological information from noninvasive tools.^138^ Scaling up optical recordings to larger animals requires the development of faster microscopes with wider fields of view (FOV) combined with optical access in highly scattering brain tissue. Typically, LSMPM, the most popular tool for depth-resolved imaging of calcium signals, can scan a ∼600×600  μm xy plane with submicrometer resolution in ∼0.5  s when using regular galvo scanners. Collecting a stack of such planes by physically moving the focal plane by increments of ∼2  μm to create a 3D image that is ∼600  μm deep thus requires tens of seconds, which precludes volumetric measurement of calcium dynamics. Thus, the trade-off between temporal resolution, spatial resolution, and FOV of standard LSMPM is limiting recordings of neuronal calcium to two-dimensional (2D) subregions in the mouse brain. With the goal of bridging the study of neuronal microcircuits to that of whole brain dynamics, several groups are pushing these boundaries by developing ever faster and larger FOV microscopes.

Technical advances in optical engineering are key towards this goal. For example, an 8×10  mm FOV two-photon microscope with 1  μm lateral resolution and 5 mm/ms scan speed was developed using large diameter compound lenses to minimize aberrations.^139^ In classical laser-scanning designs, faster z-scanning can be achieved using a piezo-electric driven objective; in the transverse (xy) plane, resonant galvanometer mirrors can be used to increase scan rates tenfold. Furthermore, fast volumetric imaging can be achieved by bypassing entirely the need to scan the laser excitation beam in one or more dimensions. Lightsheet microscopy uses a 2D layer of illumination perpendicular to the objective to image an entire xy plane at once but requires sample transparency to image large volumes. Its use to image whole brains in mice has thus been limited to *postmortem* preparations using optical clearing agents,^140^^–^^142^ but implantable photonic probes that can image restricted FOV *in vivo* are also being developed.^143^ Alternatively, one could bypass the z scanning direction *in vivo* with LSMPM by extending the focal point of the excitation beam axially to obtain a focal line. The resulting so-called Bessel focus allows the simultaneous excitation of fluorophores spanning a depth of several tenths of microns, albeit without depth resolution.^144^

Multidisciplinary technical developments have allowed the activity of a growing number of neurons to be simultaneously recorded in the mouse cortex. Kim et al.^145^ have for instance developed large curved cranial windows that fit most of the cortical surface, granting optical access to ∼ a million neurons and allowing them to measure from volumes comprising ∼10,000 neurons with sufficient spatial and temporal resolution. Using a Bessel focus, Lu et al.^146^ reported imaging a 301×450×612  μm volume at 3.2 Hz and recording 9247 active inhibitory neurons within a 3020×1500×600  μm volume at 1 Hz. Using resonant LSMPM, calcium traces of 16,000 neurons were observed over a 3-mm square of the mouse cortex scanned at 7.5 Hz.^147^ Another group reported calcium recordings of ∼10,000 neurons at 2.5 Hz spanning 11 imaging planes spaced at 35  μm.^148^ Such datasets, which lie in very high-dimensional spaces, have been studied from various angles using data clustering and dimensionality reduction techniques.^149^^–^^151^

Despite the technical and computational challenges associated with the relatively large scale of neuronal dynamics in rodents, they remain one of the most promising models for translating results to human brains. This is due in part to their relative phylogenetic proximity as mammals, combined with their physical size appropriate for both cellular calcium imaging as well as noninvasive BOLD-fMRI. Such a combination of invasive microscopic with noninvasive macroscopic imaging within a same species offers a unique opportunity for translational neuroscience.^138^ Mostly due to their larger brains, rats remain the most widely used species in fMRI studies^95^^,^^152^ and have been used to obtain concurrent fMRI and optical measurements for more than a decade.^153^^–^^155^ Since then, the use of mice for fMRI studies has also been steadily increasing,^152^^,^^156^ with combined optical recordings having recently been demonstrated within the same individual^157^ and even simultaneously.^158^ For the study of brain networks, the possibility of combining whole-brain fMRI signals with publicly available mouse atlases of structural connectivity and gene expression from the Allen Institute^19^^,^^159^ makes mice an especially interesting model. Over the past decade, there has also been an explosion in the number of available transgenic mice lines and genetic tools, further contributing to the wide adoption of this model to answer various neuroscience questions (see Ref. 160 for a review of available tools). Here, we focus on the imaging—rather than manipulation—techniques, reviewing combinations of all-optical or MR-optical methods that allow us to examine the interactions between neurons and vessels across scales.

### 4.2 Toward a Description of Neurovascular Dynamics at the Microscopic Scale

The study of the coupling between neuronal, metabolic, and vascular activity has been an active field of research in recent decades. Much effort has been devoted to trying to understand the link between macroscopic BOLD signals and electrophysiology in specific frequency bands,^34^^,^^161^^,^^162^ which is notably difficult because of the high regional variability of this relationship.^163^ On the other hand, another active area of study on neurovascular coupling involves a search for the molecular pathways that allow cells of the NVU to coordinate blood flow (see reviews in Refs. 2, 164, and 165). Recent developments in imaging methods and genetic tools now also make it possible to observe how CBF is dynamically regulated by the NVU at the smallest scales of the cerebrovascular network.^166^ The typical approach for this requires to simultaneously, or under the same conditions, measure activity in both specific NVU cells and blood vessels.

At the microscopic, single-cell, and single-vessel scale, LSMPM has made its way as the imaging tool of choice. By spectrally separating emitted fluorescence from multiple contrast agents, LSMPM can simultaneously measure multiple contrasts during one experiment, for example, to combine GECIs with intravascular injections of blood plasma dyes^167^ to simultaneously observe activity in NVU cells and vessel diameter or red blood cell velocity. *In vivo* optical access in such experiments is usually provided from either a thinned skull preparation or a craniectomy sealed with a glass window.^168^ This approach allows researchers to take full advantage of the cellular specificity that GECIs provide to study neurovascular interactions in multiple NVU cells, for example in pericytes,^169^ astrocytes,^170^ microglia^171^ or even in specific neuronal populations simultaneously.^172^ GECI expression has typically been achieved in transgenic mice lines or through invasive AAV virus injections directly into the brain, but the advent of AAVs that can cross the blood–brain-barrier and thus be intravenously injected^173^ now makes this tool even more accessible. Calcium indicators can further be combined with the cell-type-specific targeting of channelrhodopsins to perform optogenetic stimulation to causally study the cellular specificity of neurovascular coupling using all-optical methods.^174^ Many such optogenetic studies are conducted at the mesoscale using widefield imaging, which we discuss later.

**Fig. 2:**
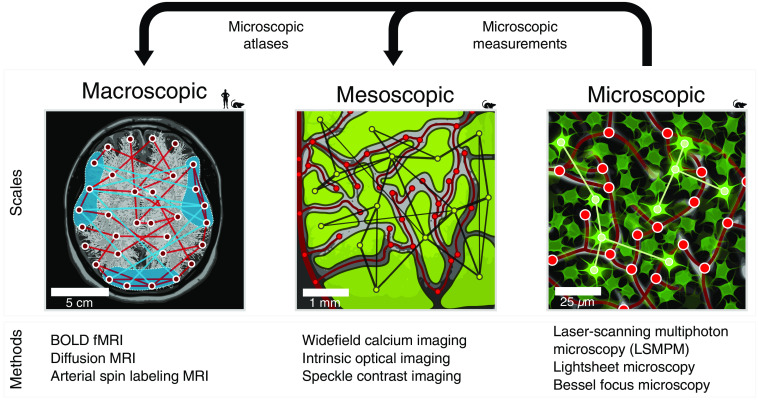
Imaging neurovascular networks at different spatial scales. The interactions between neurons and vasculature can be observed *in vivo* at different spatial scales. At the microscopic scale, calcium imaging in neurons at cellular resolution can be combined with colocalized imaging of blood vessels and other cell types within the NVU. At the mesoscopic scale, widefield calcium imaging can be combined with vascular optical measurements in cortical surface vessels. At the macroscopic scale, BOLD fMRI measures entangled neuronal and vascular interactions. Structural connections are measured using diffusion MRI and regional blood flow using arterial spin labeling. Small pictograms depict the translational perspective of macroscopic noninvasive brain imaging, whereas smaller scales are only accessible in animal models. Bridging across scales can be accomplished experimentally with multiscale measurements in a single animal. Compiling results from standardized measurement protocols repeated across entire brains can yield statistically representative maps of microscopic properties, or atlases, to which macroscopic datasets can be coregistered.

Using simultaneous blood vessels and cellular markings with LSMPM has recently allowed researchers to study how blood flow is regulated in microscopic networks of capillaries. Recent work has indeed revealed that CBF is not only controlled at the level of large descending arterioles through vascular smooth muscle cells to uniformly feed large populations of neurons but also that even within small microvascular networks, calcium-dependent mechanisms allow small groups of neurons to finely tune blood flow distribution on a branch-to-branch basis.^175^ At this level, pericytes and endothelial cells communicate via gap-junctions according to a connectivity map that is locally modulated by activity to propagate focal vasomotor responses in a preferred direction along a capillary branch.^176^ At branching points of a capillary circuit, single junctional pericytes extend processes over splitting branches that can differentially contract to direct flow in one branch or another depending on the location of activity.^177^ In the mouse retina, even remote, nonadjacent capillary branches have been shown to communicate to finely allocate blood according to activity levels via pericytes that possess nanotube-like structures through which vasoconstrictive signals can travel.^178^ Different nonstructurally connected regions of the vasculature may thus effectively form functional connections via the intermediary of pericytes. This body of work suggests a view of the microvasculature as not just a passive blood distributing structural network, but as a functional network dynamically engaged in controlling blood flow. Recent studies have also proposed that different layers of this network may modulate CBF on distinct characteristic time scales, with capillary pericytes exerting a slower constricting effect than mural cells on larger upstream vessels.^176^^,^^179^ Similarly, studies hint that astrocytic CBF regulation may act on a faster time scale and through different molecular pathways at the capillary level than on larger upstream arterioles.^111^^,^^170^^,^^180^^,^^181^ These observations illustrate that blood flow regulation in the brain is achieved by diverse functional components (i.e., NVU cells) of a rich vascular network acting at multiple spatial and temporal scales. A full understanding of neurovascular interactions thus involves the study of neuronal and vascular networks across multiple scales (Fig. 2).

Future studies combining fast, large FOV imaging with cellular and single-vessel resolution recordings open the door to obtaining experimental datasets of larger-scale neurovascular networks. For example, a Bessel focus LSMPM was employed to monitor vascular dilation in individual vessels at volumetric video rate,^182^ which could be simultaneously imaged with neuronal calcium activity in the same volume.^146^ Another modality promising for fast volumetric imaging of vascular dynamics is optical coherence tomography (OCT). OCT is not based on fluorescence but rather on intrinsic refraction-based contrast and measures depth-resolved signals using spectral information—again bypassing the need for scanning in the z direction. It can be used for structural microangiography and dynamic flow measurements using Doppler OCT.^183^ OCT could be combined with fluorescence techniques for simultaneous calcium and vascular imaging. Some advantages of OCT over fluorescence microscopy are that it does not require tracer injection, that wavelength (thus maximal imaging depth) can be selected without worrying about fluorophore excitation spectra, and that volumetric scanning is achieved by 2D scanning of the illumination.

Despite such continuous technical development to transcend the FOV and time resolution constraints of LSMPM, the recording of multiple regions spanning large FOV necessary to study meso or macroscale brain connectivity currently requires the use of other imaging techniques which cannot achieve microscopic, single-cell resolution.

### 4.3 Scaling Up: Toward Translational Measurements of Neuronal and Vascular FC

For assessing both the neuronal and hemodynamic components of large-scale FC at a cortex- or brain-wide scale, measures of neuronal and vascular signals can be combined in mesoscale imaging systems. To achieve large FOV, calcium indicators can be imaged with widefield systems, albeit without depth resolution. In these systems, GCaMP is excited over the entire FOV (typically using an LED) and fluorescence is measured with a camera,^184^ forming images all at once as opposed to the point-by-point approach of LSMPM. Widefield imaging can be performed through the intact or thinned skull as well as through transparent cranial windows, although the latter may limit the size of the observable region. For this reason, window preparation and implantation methods have been optimized to provide large FOV,^145^^,^^168^^,^^185^ and it was demonstrated that cranial windows used for optical imaging are compatible with MRI.^157^ In addition to fluorescence signals, widefield systems can measure wavelength-specific reflectance to report on large-scale cortical blood volume and oxygenation, a technique called intrinsic optical imaging.^186^ The principle is to estimate concentration changes in oxyhemoglobin and deoxyhemoglobin, whose absorption spectra are known, from changes in the intensity of reflected light at multiple wavelengths. Hemodynamic signals have a direct artifactual influence on calcium fluorescence measurements, as emitted fluorescence will be partially absorbed by blood and its intensity will covary with blood volume. Relatively easy to implement methods have, however, been proposed to correct this artifact.^187^^,^^188^ Widefield calcium and intrinsic optical imaging can in addition be combined with blood flow measurement techniques, such as laser speckle contrast imaging, to create versatile neurovascular imaging systems that can simultaneously measure cellular calcium, blood oxygenation and volume, and CBF.^157^^,^^184^

One advantage of widefield neurovascular imaging is that it can easily be combined with optogenetics to causally study the role of various cell-types in shaping cortical hemodynamics.^188^ By specifically expressing the light-gated cation channel channelrhodopsin, a light source can be used to depolarize a group of targeted cells while the widefield system measures the associated hemodynamic signals. A promising research avenue that takes advantage of this methodology aims to identify cell-type-specific hemodynamic signatures that could eventually enable noninvasive measurement techniques such as fMRI to be used to infer activity in different neuronal populations, effectively bridging the scale from macroscopic to microscopic brain imaging.^189^^,^^190^ Widefield imaging systems and optogenetics have been used to compare the vascular outcomes of optically activating neurons and astrocytes,^191^ excitatory and inhibitory populations,^192^^–^^195^ and multiple subtypes of inhibitory neurons.^196^ Although most of these studies do not converge yet toward a consensus that could clearly allow to disambiguate the activity of specific neuron-types in BOLD signals, a recurrent observation important for BOLD fMRI is that different subtypes of interneurons can inhibit excitatory activity while producing both vasodilation and vasoconstriction, often in sequence, resulting in biphasic CBF responses.^157^^,^^174^^,^^193^^,^^194^^,^^196^

Another new key imaging opportunity for the study of neurovascular brain circuits at the neuronal population scale is that of laminar fMRI measurements. The development of ultrahigh-field (≥7  T) scanners and new MRI sequences, including non-BOLD sequences such as CBV-weighted vascular space occupancy, now provide researchers with enough spatial resolution to resolve individual layers of the cortex in whole brain fMRI scans.^197^^–^^200^ From a network perspective, studying laminar signals can add another dimension to FC as each cortical brain region can be decomposed into its constituent layers, those layers often being known to contribute to information flow in specific directions. For example, tract tracing studies show that most feedforward thalamocortical connections end in L4, while corticothalamic projections originate from L5 and L6,^201^ those from L5 being considered feedforward and those from L6 feedback.^202^ Based on hypotheses inferred from such anatomical data, one can use laminar-specific fMRI to add a directionality element to FC in humans.^197^^,^^203^ One can also use layer-fMRI to verify predictions from computational theories of brain function, which often assign specific roles to neurons in different layers.^204^

Cortical layers possess distinct vascular features that are necessary to consider when analyzing laminar fMRI data. For example, the average orientation of capillary segments strongly varies with cortical depth, as capillary branches only have weak orientation preference in deeper layers but become more and more normal to the pial surface in superficial layers.^205^ Such anisotropy has been shown to have profound effects on MRI signals.^206^^,^^207^ Microvascularization levels also vary between layers, capillary volume density being highest in intermediate layers and lowest in L1,^104^ which makes the laminar fMRI signal unavailable from this layer.^197^ These examples show that a reliable interpretation of high-resolution fMRI requires a detailed understanding of the brain’s vascular architecture at this scale (see Sec. 4.5).

**Fig. 3:**
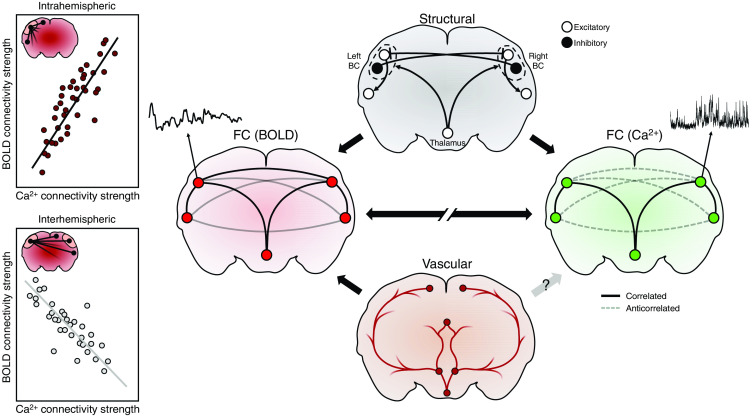
Divergence between BOLD FC and neuronal FC. Although a good agreement is generally observed between FC derived from BOLD and from calcium measurements, the cell-type specificity of neurovascular coupling can potentially lead to divergence between the two, as observed in Ref. 158. Specifically in the mouse barrel cortex, the expected positive relationship between connectivity strength evaluated with BOLD and excitatory calcium signals holds only when considering interhemispheric nodes (left upper panel). When considering intrahemispheric nodes, the relationship is negative (left lower panel). Taking into account the likely presence of strong interhemispheric inhibition in the barrel cortex, this discrepancy can be explained by positing that inhibitory populations can simultaneously generate positive BOLD signals while inhibiting excitatory neurons across hemispheres. The arrows in the right panel represent the relationships between the different networks, with hemoneural interactions possibly mediating a link between the vascular and functional neuronal networks. The left panel is a sketch of the results from Ref. 158.

To study functional networks at the scale of the entire cortex, several groups have combined widefield calcium and hemodynamic imaging, either using intrinsic optical imaging^184^^,^^208^^–^^211^ or BOLD fMRI.^158^^,^^212^ Although these studies have mostly shown a good agreement between calcic- and hemodynamic-based FC, some departures have also been observed. For example, Lake et al. observed that within a given hemisphere, FCS in the mouse barrel cortex measured with BOLD was positively correlated to FCS measured with calcium signals from excitatory neurons.^158^ However, when only interhemispheric connections were considered, higher BOLD FCS was associated with lower calcium FCS. To explain this discrepancy, the authors hypothesized that interhemispheric inhibition could simultaneously be decreasing excitatory activity while increasing BOLD signals (Fig. 3).

This interpretation of their results highlights the importance of being equipped with a good model of neurovascular coupling (furthermore, of cell-type specific coupling) to explain how BOLD connectivity results from neuronal interactions. Lake et al.’s hypothesis is based on the aforementioned studies on neuron-type-specific neurovascular coupling that demonstrate how inhibitory neurons can simultaneously decrease overall neuronal activity and increase blood flow by producing vasoactive compounds.^196^^,^^213^^,^^214^ To reliably test such hypotheses about the physiological mechanisms behind the BOLD effect, generative models of neurovascular signals, i.e., models that try to explain how a neuronal signal (e.g., synaptic activity) is eventually transduced to a vascular one (e.g., vasodilation), provide a powerful and necessary tool. The recent notion of cell-type-specific neurovascular coupling has indeed begun to be formulated in such model form.^215^ However, neurovascular models typically describe local interactions between neurons and vasculature in singled-out brain regions. Since we are concerned with the task of explaining BOLD FC, generative models also need to be embedded within models of connectivity, i.e., models that can account for the effects that different regions exert on each other.

### 4.4 Getting Causal: Modeling Functional Connectivity

Models that try to predict connectivity patterns in a feedforward (generative) manner are vital to our ability to answer questions about the neurovascular origins of BOLD FC, but also allow us to bring the study of brain connectivity a step further. By definition, FC is an observational measure of statistical dependency between nodes and as such cannot be used to infer causality (directionality) in the links between them. The causal influence that different regions exert on each other is captured by a distinct concept called effective connectivity (EC).^216^ EC and FC are related, since effective connections dictate which (not necessarily connected) regions can become partially synchronized (functionally connected). A typical example of divergence between FC and EC is the case when two physically unconnected brain regions receive a common input that drives them toward synchronization.

EC can be inferred from generative models of brain signals (measured with fMRI, EEG, or other techniques). One of the most prominent frameworks of this kind is DCM.^24^^,^^25^ DCM is used to estimate EC between specified brain regions by first forming a generative model of BOLD data that mathematically describes how neuronal signals are translated to hemodynamic and then to BOLD signals. To represent interregional interactions, neuronal activity is modeled with coupled differential equations (one for each of the regions considered), in which EC is introduced in the form of a coupling matrix that mediates the strength of the interactions between regions. By fitting the generative model to the measured BOLD regional time-series using Bayesian inference, model parameters of interest are estimated. These include the EC matrix, which can then be used to uncover effective brain networks and their graph-theoretical properties,^217^ but also modeled neuronal and vascular parameters. DCM is thus a powerful framework to separate the underlying neuronal and vascular correlates of measures of brain-wide distributed BOLD activity under the assumptions of the chosen model.

The question of choosing a proper generative model is hence key to interpreting EC. To this end, DCM is used in combination with Bayesian model comparison to choose between different models according to their relative statistical evidence.^218^^,^^219^ This way, one can use DCM to explain FC according to the basic tenets of the scientific method, starting by observing a pattern (BOLD FC), generating multiple hypotheses attempting to explain that pattern (EC + generative model) and systematically evaluating and comparing the evidence for each of these hypotheses (Bayesian model comparison). Such a procedure can be used both at the local level, for example, to evaluate if neurovascular coupling is best described by synaptic input or spiking output,^220^ and at the network level to evaluate how different neurovascular coupling models impact estimates of EC.^221^ Based on the ideas we have presented here, one could also test whether including vasculoneuronal interactions in a model helps predict observed network dynamics in a specified context, for example, during vascular disease-induced prolonged reductions in CBF.

Although DCM is typically used with human neuroimaging data, animal studies provide the means to directly access modeled hidden (neuronal and vascular) signals to inform model selection. This idea has, for example, been used in humans to merge EEG and fMRI data,^222^ but invasive animal studies using new multimodal imaging tools can go a step further by providing both spatially and temporally resolved neurovascular measurements^223^ (Fig. 2). As we have emphasized, these also offer the exciting opportunity to explore the multiscale and multilayered aspects of brain networks^224^^,^^225^ and thus to ask questions about how neurovascular network properties at the cellular scale (neuronal/capillary microcircuits) translate to observations at larger scales (neuronal populations/large vessels).

With the above example of DCM, we show how vascular measurements can be embedded into a computational modeling framework to generate causal interpretations of neuronal dynamics, but this idea can further be extended to other modeling approaches. Artificial neural networks have become a widely used tool in the study of real neuronal systems.^226^ Of particular interest, recurrent neural networks, through their rich internal dynamics, are especially well-suited to model neurobiological dynamical systems.^227^ They can be trained to reproduce real-time series data, and the trained network can be reverse engineered to infer mechanisms of interaction between network elements.^228^^,^^229^ Multilayer approaches can also model different interacting compartments of a system, such as neuronal and astrocytic layers^230^ but also vascular layers which impose energetic constraints^231^ or interact bidirectionally^232^ with neuronal layers.

As opposed to models of dynamical systems based on differential equations, another prominent approach in network neuroscience is the descriptive study of network topological properties without any assumption about a generative model. We have argued in this article that vascular and neuronal systems form an overlapped and even possibly entangled neurovascular network. For the observational use of BOLD FC, we have shown how incorporating vascular measurements with BOLD could add a meaningful dimension to characterize brain networks in health^65^ and disease.^74^^,^^75^ Similarly, models that aim to quantify the relative associations between various physiological properties and BOLD FC would benefit from the incorporation vascular properties. Linear models and correlations have been predominant in the study of the structure–function relationship of brain networks,^233^ suggesting many principles by which structural networks could explain variance in functional interactions, notably through indirect pathways or diffusion-like processes.^234^ Earlier studies assumed homogeneity in SC-FC coupling across network nodes, but the recent recognition of the spatial variability of this relationship^137^^,^^235^ points to the importance of taking regional properties and topological embedding into consideration. A recent review on the structure–function relationship by Suárez et al.^236^ draws the attention on biologically informed models where microscopic regional inhomogeneities in laminar differentiation and cytoarchitecture^237^ or gene expression^238^ are considered as node properties, yielding better predictions of FC. Network analyses will certainly benefit from a growing effort in developing large databases of microscopic properties distributed over macroscopic scales, which are discussed in Ref. 239. In line with the idea of considering vasculature not as a confound but as a potential predictor of network activity and connections, we ask whether including regional vascular properties or blood flow across regions would add significant depth to macroscale network analysis. Rodent studies will once again be key to test this idea, as recent developments are now allowing the measure of microvascular structure at the whole brain scale in mice, from which vascular graphs and their properties can be extracted.

### 4.5 Measuring Structural Vascular Networks

The brain vascular system is naturally organized as a network of interconnected segments with varying morphological and mechanical properties matching their different functional roles. The topology of this network varies along the vascular tree (from pial to penetrating vessels and capillaries) and has been well characterized, as reviewed in Ref. 240. To probe this topology, new *in vivo* and* postmortem* imaging techniques have facilitated the measurement of microvascular network structures in animal models, allowing 3D images of the vascular tree to be transformed into graph representations that can be used within the framework of graph theory. Automatically segmenting vascular images and creating a graph representation of them has been achieved using a machine learning approach.^241^ Efforts have also been put toward synthetizing artificial vascular networks using computational models that accurately reproduce real vascular network properties.^242^ *In vivo*, volumetric images of microvasculature can be measured using LSMPM in combination with exogenous intravascular contrast agents^167^ or genetically encoded labeling of endothelial and mural cells.^243^ The advantage of *in vivo* measurements is that they can be performed longitudinally and analyzed in a framework of dynamical networks, but they are limited by the FOV constraints of LSMPM. With *postmortem* preparations, however, the vasculature can be imaged throughout the entire brain using mechanical slicing or optical techniques.^244^

Using the former in gel-infused preserved brains, Blinder et al.^245^ obtained a graph representation of the barrel cortex vasculature, but only recently was the whole brain vasculature down to the capillary level imaged in mice. Xiong et al.,^246^ for example, used optical sectioning tomography and a modified Nissl staining method to construct a vascular atlas of the mouse brain. Quintana et al.^247^ reconstructed the whole-brain vasculature based on vascular corrosion casts imaged with microCT. In 2020, Kirst et al.^140^ presented an impressive contribution in which the entire mouse brain vasculature was imaged with lightsheet microscopy following immunolabeling and tissue clearing; a pipeline for automatically creating vascular graphs was developed and yielded the first graph representation of the entire mouse brain vascular network. Finally, Ji et al. recently obtained a whole brain microvascular connectome at submicrometer resolution using serial LSMPM imaging in lumen-perfused brains, allowing them to perform geometrical and topological analysis using precisely measured capillary radii.^205^ Such advances will be key for bottom-up modeling of functional imaging signals, for example, with the vascular anatomical network model proposed by Gagnon et al.,^206^ which uses microvascular angiograms as a basis to simulate BOLD time series. Another application is the integration of microvascular properties in brain network models, for example, to study the structure–function relationship. To this end, standardized large-scale vascular atlases could eventually be produced, taking advantage of the ever-growing open science and collective efforts agenda.^239^

## 5 Conclusion

The considerations presented in this paper have highlighted the ambiguous dual neurovascular nature of BOLD-derived FC. Instead of simply noting the various ways in which vasculature can influence BOLD neuronal representations, recent evidence allows us to propose an interpretative framework of overlapped neuronal and vascular networks to describe BOLD functional networks. Furthermore, the apparent synergy between neuronal and vascular systems suggests a view of the vasculature as not merely an additional layer between neurons and BOLD measurements, but as a component of an entangled and functionally relevant neurovascular network. Far from being a nuisance, neurovascular networks might also prove more useful than purely neuronal ones in identifying early markers of neurodegenerative diseases that are characterized by an early onset of vascular dysregulation.^113^^–^^116^^,^^248^ BOLD FC by itself has already shown promise as a source of observational markers of disease state,^41^^,^^42^^,^^44^^,^^249^ but distinct clinical populations can remain hard to differentiate.^47^ Using methods that can further distinguish the two components of neurovascular networks opens the door to the discovery of new disease biomarkers in humans,^75^ and in combination with invasive imaging in translational animal models and appropriate modeling frameworks, will eventually allow us to move toward a more causal understanding of network disruption in disease. Given that neurovascular interactions can be observed at multiple scales, this process will be helped by multiscale and multimodal imaging data that will facilitate the complementation of macroscale imaging observations with microscopic measurements, enabling us to paint a more complete picture of brain networks and their alteration in disease.
